# Characterization of autoimmune eye disease in association with Down's Syndrome

**DOI:** 10.21203/rs.3.rs-2766021/v1

**Published:** 2023-05-03

**Authors:** James Rosenbaum, Amr Zaki, Sirichai Pasadhika, Jerry Huang, Akshay Thomas, Bryn Burkholder, Lyndell Lim, Stephanie Llop, Eric Suhler, Grazyna Adamus

**Affiliations:** Legacy Devers Eye Institute; Keelung Chang Gung Memorial hospital, Keelung

## Abstract

**Background:**

Autoimmunity and deficiency of the transcription factor autoimmune regulator protein (AIRE) are known associations with Down Syndrome (DS). Lack of AIRE abrogates thymic tolerance. The autoimmune eye disease associated with DS has not been characterized. We identified a series of subjects with DS (n = 8) and uveitis. In 3 consecutive subjects, we tested the hypothesis that autoimmunity to retinal antigens might be a contributing factor.

**Subjects/Methods:**

This was a multicentered, retrospective case series. De-identified clinical data of subjects with both DS and uveitis were collected via questionnaire by uveitis-trained ophthalmologists. Anti-retinal autoantibodies (AAbs) were detected using an Autoimmune Retinopathy Panel tested in the OHSU Ocular Immunology Laboratory.

**Results:**

We characterized 8 subjects (mean age 29 [range, 19–37] years). The mean age of uveitis onset was 23.5 [range, 11–33] years. All 8 subjects had bilateral uveitis (p < 0.001 based on comparison to published university referral patterns), with anterior and intermediate uveitis found in 6 and 5 subjects respectively. Each of three subjects tested for anti-retinal AAbs was positive. Detected AAbs included anti-carbonic anhydrase II, anti-enolase, anti-arrestin, and anti-aldolase.

**Discussion:**

A partial deficiency in the AIRE on chromosome 21 has been described in DS. The similarities in the uveitis presentations within this patient group, the known autoimmune disease predisposition in DS, the recognized association of DS and AIRE deficiency, the reported detection of anti-retinal antibodies in patients with DS in general, and the presence of anti-retinal AAbs in 3 subjects in our series supports a causal association between DS and autoimmune eye disease.

## INTRODUCTION

Down syndrome (DS) is a chromosomal disorder that is caused by the presence of an extra genetic copy of chromosome 21. Trisomy 21 is the most common form of DS wherein there are 3 separate copies of chromosome 21 within the affected individual’s genome. Another form of DS involves the partial translocation and attachment of chromosome 21 onto another chromosome. A milder and rare form of DS resulting from mosaicism also exists^1^. Trisomy 21 and translocational DS are clinically identical and are associated with physical growth and developmental delays, mild to moderate intellectual disability and characteristic physical features including upward slanting of the palpebral fissures, nasal bridge flattening, brachycephaly and hypotonia. Systemic complications include congenital heart disease, gastrointestinal malformations, deafness, atlanto-axial dislocation and myeloproliferative disease ^1-3^.

Higher rates of autoimmune disorders such as diabetes mellitus^3-5^, celiac disease^6-8^, thyroid dysfunction^9^ and inflammatory arthritis ^10-12^ are present in patients with DS. AIRE (autoimmune regulator) is a transcription factor expressed by thymic epithelium. It allows the thymus to delete auto-reactive lymphocytes to prevent the development of autoimmunity. The absence of AIRE results in a disease called autoimmune polyglandular syndrome 1 (APS-1) which is characterized by multi-organ autoimmunity, including uveitis^13^. Patients with DS have reduced AIRE expression^14^. A paper published in *Nature* in 2023 documented many immunologic abnormalities in patients with DS including an elevation of numerous cytokines, elevated IL-6 signaling in CD4 positive T cells, autoreactive B cells, and 365 different autoantibodies^15^. The same study detected only 257 autoantibodies in serum from patients with APS-1. Many of the autoantibodies detectable in patients with DS are directed at antigens found in the central nervous system. One such antigen is ATP1B2, which is also expressed in the retina^16^.

Although various ophthalmological findings including high refractive error, nystagmus, strabismus, lacrimal system abnormalities, blepharitis, cataracts and persistence of foveal inner retinal layers have been shown to be more prevalent with DS ^17-20^ uveitis or autoimmune eye disease in this patient group is not well characterized. Given the reduction in AIRE protein and the higher prevalence of autoimmune and inflammatory conditions in these patients, our aim was to characterize the autoimmune eye disease in patients with DS. We reasoned that if the inflammatory disease demonstrated consistent phenotypic features, the argument that the immune-mediated disease was causally related to DS would be strengthened. In addition, we were able to test a subset of consecutive subjects for the presence of anti-retinal AAbs.

## METHODS

After identifying 4 cases in Portland, Oregon (2 at Casey Eye Institute and 2 at Devers Eye Institute), a request to share similar cases was posted on the American Uveitis Society and Proctor Foundation listservs. A multi-centered retrospective chart review was performed and de-identified data were collected via an Institutional Review Board (IRB)-approved questionnaire completed by uveitis specialists. All clinical data were collected prior to June 30, 2020. IRB approval was granted by Oregon Health & Science University. Anti-retinal antibodies were tested in the OHSU Ocular Immunology Laboratory as previously described ^21,22^.

## RESULTS

**Figure 1:**
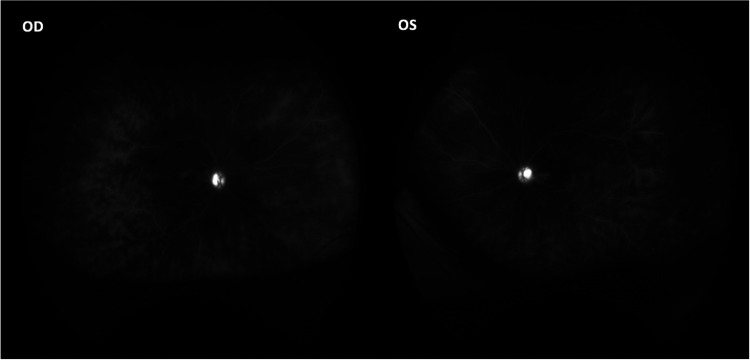
Optos fundus fluorescein angiography of subject 7 showing bilateral optic disc hyperfluorescence and diffuse vascular leakage.

The data collected for each of the 8 DS subjects in this retrospective case series are presented in a set of four tables. Subject demographics and general uveitis characteristics are shown in Table 1. This table provides information on the patient age, gender, bilaterality of eye disease, and initial diagnosis. All 8 subjects presented with simultaneous bilateral uveitis with anterior and intermediate involvement found in 6 and 5 subjects respectively. One subject presented with findings of bilateral posterior uveitis (multifocal choroiditis) and another subject presented with only retinal vasculitis and cystoid macular edema (CME) (Fig. 1).

Table 2 shows the ocular complications of the eye disease and makes clear that many of these patients shared a similar phenotype. Table 3 provides additional information on glaucoma in particular. Six of 8 subjects (75%) had been on immunomodulatory therapy for treatment of their uveitis (Table 4).

**Figure 2:**
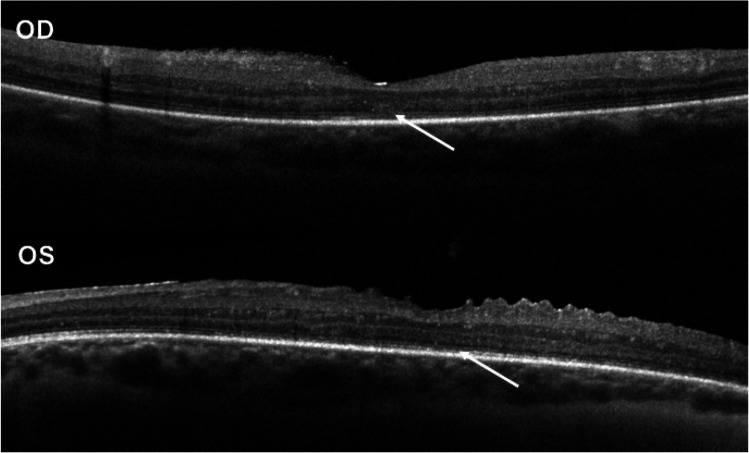
Spectral domain-optical coherence tomography (SD-OCT) of subject 8 showing bilateral ellipsoid zone disruption (white arrows).

Macular optical coherence tomography (OCT) imaging was obtained on 5 subjects one of whom had ellipsoid zone disruption (Fig. 2).

Three subjects were available to be tested for anti-retinal AAbs. Subject 5 had AAbs against carbonic anhydrase II (CAM), aldolase, enolase, arrestin, and glyceraldehyde 3-phosphate dehydrogenase (GAPDH). Subject 7 had anti-CAII AAbs and subject 8 had anti-enolase alpha AAbs (Table 1).

## DISCUSSION

DS is the most common chromosomal disorder causing mild to moderate intellectual disability. DS can be due to Trisomy 21, translocation of chromosome 21 or mosaicism^1^. Although ocular manifestations have been described in patients with DS,^17-20^ uveitis has not been characterized in this patient population to our knowledge.

In this case series of subjects with DS and uveitis, the mean onset of uveitis occurred in the third decade of life and 7 out of 8 subjects were diagnosed with idiopathic uveitis. The majority of subjects (6 of 8) had anterior involvement with 5 also having an intermediate component. One subject presented with posterior uveitis in the form of multifocal choroiditis (not associated with anterior chamber cell or vitreous cell). One subject had retinal vasculitis and CME with no documentation of anterior or intermediate uveitis. This subject was followed by a general ophthalmologist for a year prior to referral to a uveitis specialist and given the lack of detailed early records, we cannot exclude prior anterior or intermediate involvement. Since two of the described subjects did not have a documented leukocytic infiltrate, since at least one subject had ellipsoid zone loss detected on OCT, and since autoantibodies seem likely to play prominent role in this disease (see discussion below), the term autoimmune eye disease might be more broadly appropriate than the term, uveitis. We prefer to use the term, uveitis, if a leukocytic infiltrate is present in the anterior chamber or the vitreous humor. A few diseases like acute zonal occult outer retinopathy (also known as AZOOR) are often described as a uveitis despite the absence of a leukocytic infiltrate. In contrast, an autoimmune retinopathy is usually associated with a relative lack of infiltrating leukocytes and often ellipsoid zone loss. The ocular inflammatory disease associated with DS has aspects of uveitis and autoimmune retinopathy, in keeping with the observation that both anti-retinal antibodies and an autoimmune diathesis are characteristic of patients with DS. All the subjects in this study had bilateral involvement at the time of uveitis diagnosis. Cataract development (7 of 8 subjects) and ocular hypertension/glaucoma (7 of 8 subjects) were also common features in this subject group.

The bilaterality of the anterior uveitis presentations in this cohort is an interesting finding given that unilateral presentations are more common in the general population. A prospective study by McCannel *et al*., showed that 9.8% of anterior uveitis cases evaluated by community-based practices were bilateral compared to 32.6% in university referral practices^23^. Using the more conservative measure of bilaterality from university-based practice, the likelihood for 8 consecutive subjects all to be bilateral is (0.326)^8^ or p < 0.001. Similarly, the frequency of glaucoma, cataract, relatively similar age of onset, and severity prompting immunosuppression support the concept that indeed this is a distinct entity.

As noted in the introduction, patients with DS have higher rates of autoimmune conditions including diabetes mellitus^3-5^, celiac disease^6,7^, thyroid dysfunction^9^ and inflammatory arthritis^10-12^. A possible explanation for these increased rates may be due to a higher incidence of immune system dysregulation. Huggard *et al*., showed that children with DS have increased levels of inflammatory and anti-inflammatory cytokines when compared to age-matched controls; these cytokines include interleukin (IL)-1β, IL-2, IL-6, IL-10, IL-1 receptor antagonist (RA), erythropoietin (EPO), vascular endothelial growth factor (VEGF) and granulocyte-macrophage colony-stimulating factor (GM-CSF)^15,24,25^. Tumor necrosis factor (TNF)-α, and interferon (IFN)-γ have also been shown to be elevated in patients with DS^15,26^. Another study showed that patients with DS had lower levels of autoimmune regulator protein, AIRE, a transcription factor located on chromosome 21, resulting in reduced expression of peripheral antigens in the thymus. This phenomenon potentially leads to a failure of central tolerance and a subsequent autoimmune predisposition^14^. In fact, AIRE deficient mice are a well characterized model of uveitis due to an autoimmune response to interphotoreceptor retinoid binding protein (IRBP)^27^. Malle and colleagues found that activation of the phosphorylated STAT-3-IL-6 pathway was frequent in patients with DS ^15^. Our report is a retrospective study on a small number of subjects. As such, we did not attempt to compare therapeutic interventions. However, after the preparation of this manuscript, we treated one DS subject with tocilizumab, a solute IL-6 receptor. She developed macular edema following cataract surgery and responded extremely well to the inhibition of IL-6.

AIRE deficiency in humans results in a syndrome called APS-1, autoimmune poly-glandular syndrome. In a series of 91 patients from Finland with APS-1, 6 had iridocyclitis and 2 had retinal dystrophy^13^. Some of our subjects could be incorrectly diagnosed with a retinal dystrophy due to irregularity in the ellipsoid zone of the retina on OCT (optical coherence tomography) imaging and a relative lack of a cellular infiltrate in the uveal tract.

Patients with DS are considered a vulnerable population such that medical research involving patients with DS should adhere to strict regulation. This appropriate ethical standard made it more difficult to test for anti-retinal antibodies in subjects who resided outside of the Pacific Northwest. We had access to only 3 sera, each of which tested positive. About 10 percent of healthy adults do have detectable anti-retinal antibodies ^21,22^. To have 3 consecutive subjects test positive coincidentally would be expected at a rate of (0.1)^3^ or one in a thousand. Subjects in this study were not tested for antibodies to ATP1B2, a retinal antigen and the target of an immune response in patients with DS as reported elsewhere^15^. ATP1B2 is a membrane bound protein that anchors a protein called retinoschisin.^16^ Antibodies to ATP1B2 are not detectable with the methodology used by the OHSU Ocular Immunology Laboratory.

A limitation of our study is that the association between uveitis and DS could result from chance since DS is common among chromosomal abnormalities. However, the predisposition to autoimmune conditions in patients with DS and the similarity in uveitis presentations seen in this case series, with regard to age of onset, chronicity, complications such as cataract and glaucoma, bilateral anterior and intermediate findings, and severity such that immunosuppressive therapy was common all suggest a real association between DS and uveitis. This association is made more plausible by the known deficiency of the AIRE protein, the detection of anti-retinal AAbs in the 3 consecutive subjects who were tested, and the autoimmune diathesis characteristic of patients with DS^15^.

Our series should not be considered an epidemiologic study. There are multiple reasons why an ophthalmologist might prefer to not participate in a survey study such as ours. Portland, OR, USA is a metropolitan area of about 2 million people. In 2010, the population prevalence of DS in the US was estimated to be 6.7 for every 10 000 people^28^, meaning that about 1 300 people should be living with DS in Portland metropolitan area. The US prevalence of uveitis is about 1.2 per 1 000^29^. This would mean that one or two individuals with DS in the Portland area would be expected to have uveitis. And this is potentially an overestimate since many of the DS patients would be too young for the typical onset of uveitis. Our two participating practices in Portland evaluate only a small fraction of the population in Portland, which has four large, competitive health care systems. Thus, our experience in Portland where four subjects were identified leads us to believe that uveitis is not just characteristic in presentation in DS; it is also probably increased.

In summary, although autoimmune diseases are associated with DS, the autoimmune eye disease has not been previously characterized. The recognition of this association has implications regarding pathogenesis and predicting prognosis. We hypothesize that the recognition of this association will lead to improvements in therapy based on understanding the immunologic abnormalities associated with DS.

## Figures and Tables

**Table 1:** Patient Demographics and General Characteristics

Subject	1	2	3	4	5	6	7	8
**Chromosomal Abnormality**	Translocation of chromosome 21	Trisomy 21	Unknown	Trisomy 21	Unknown	Unknown	Trisomy 21	Trisomy
**Sex**	F	F	M	M	F	F	F	F
**Age**	27	33	27	32	34	37	23	19
**Race/Ethnicity**	Caucasian	Caucasian	Indian	Caucasian	Hispanic	Caucasian	Caucasian	Hispanii
**Age at Onset**	19	32	24	11	33	31	21	17
**Uveitis Diagnosis**	Idiopathic	Idiopathic	Idiopathic	JIA-associated	Idiopathic	Idiopathic	Idiopathic	Idiopath
**Laterality of Uveitis at Diagnosis**	Bilateral (simultaneous)	Bilateral (simultaneous)	Bilateral (simultaneous)	Bilateral (simultaneous)	Bilateral (simultaneous)	Bilateral (simultaneous)	Bilateral (simultaneous)	Bilateral (simulta
**Anatomic Location of Uveitis**	Anterior + intermediate	Anterior + intermediate	Anterior + intermediate	Anterior	Anterior + intermediate	Anterior + intermediate uveitis+ anterior scleritis	Retinal vasculitis + CME	Posterio (multifo choroidi
**Uveitis Onset**	Unknown	Sudden	Insidious	Insidious	Insidious	Insidious	Insidious	Insidiou
**Course of Uveitis**	Unknown	Chronic	Chronic	Chronic	Chronic	Chronic	Chronic	Chronic
**Anti-Retinal Antibodies**	Not tested	Not tested	Not tested	Not tested	Anti-CAII, aldolase, enolase, arrestin, GAPDH	Not tested	Anti-CAII	Anti-eno alpha

F = female; M = male; JIA = juvenile idiopathic arthritis; CME = cystoid macular edema; CAII = carbonic anhydrase II; GAPDH = glyceraldehyde 3-phophate dehydrogenase

**Table 2:** Ocular Complications and Cataract History

Subject		1	2	3	4	5	6	7	8
**Macular Edema**		✓	✓	x	Unknown	Unknown	x	✓	x
**Vasculitis**		x	x	x	Unknown	Unknown	x	✓	x
**Band Keratopathy**		x	x	x	✓	x	x	x	x
**Optic Disc Edema**		x	Disc leakage on fluorescein angiography	x	x	Unknown	Disc pallor	Disc leakage on fluorescein angiography	x
**Retinal Detachment**		x	x	✓ (serous)	Unknown	x	x	x	x
**Developed Cataract?**	**OD**	✓	x	✓	✓	✓	✓	✓	✓
	**OS**	✓	x	✓	✓	✓	✓	✓	✓
**Current Lens Status**	**OD**	PCIOL	Clear	Aphakic	PCIOL	PCIOL	Cataract	Cataract	Cataract
	**OS**	PCIOL	Clear	Aphakic	PCIOL	Cataract	PCIOL	Cataract	Cataract

OD = right eye; OS = left eye; OU = both eyes; PCIOL = posterior chamber intraocular lens.

**Table 3:** Ocular Hypertension and Glaucoma History

Subject		1	2	3	4	5	6	7	8
**Developed Ocular Hypertension or Glaucoma**	**OD**	Open angle glaucoma	No	No	Open angle glaucoma	Secondary closed angle glaucoma (PAS)	Ocular hypertension (PAS)	Ocular hypertension	Ocular hypertension
	**OS**	Open angle glaucoma	No	No	Open angle glaucoma	Open angle glaucoma	No	Ocular hypertension	Ocular hypertension
**Maximum Intraocular Pressure (mm Hg)**	**OD**	45	19	NA	32	NA	35	42	25
	**OS**	40	19	NA	30	NA	15	39	25
**Current Ocular Hypertension/Glaucoma Treatment**	**OD**	Tube shunt	None	None	Acetazolamide, diode CPC	Tube shunt, brimonidine	Dorzolamide, timolol, diode CPC	Tube shunt, timolol, brimonidine, dorzolamide, latanoprost, acetazolamide	None
	**OS**	Tube shunt + timolol	None	None	Acetazolamide, diode CPC	Tube shunt, peripheral Iridotomy, brimonidine	None	Tube shunt, timolol, brimonidine, dorzolamide, latanoprost, acetazolamide	None
**Most Recent Cup-to-Disc Ratio**	**OD**	0.5	0.3	0.1	0.9	NA	NA	0.6	0.3
	**OS**	0.8	0.2	0.1	0.7	NA	0.5	0.65	0.3

OD = right eye; OS = left eye; PAS=peripheral anterior synechiae; CPC = cyclophotocoagulation.

**Table 4:** Ocular Medical and Surgical History

Subject	1	2	3	4	5	6	7	8
**Past Steroid Treatments**	Topical difluprednate, intravitreal dexamethasone implant, oral steroids	Topical difluprednate, oral steroids	Topical prednisolone, oral steroids,	Topical prednisolone	None	None	Topical prednisolone	None
**Current Steroid Treatments**	Topical Prednisolone	Topical Prednisolone	Topical difluprednate	Oral prednisone	Topical prednisolone, oral steroids	Topical prednisolone	Topical difluprednate, oral prednisone	None
**Past Immunosuppression**	Mycophenolate mofetil, cyclosporine	None	Mycophenolate mofetil, adalimumab (discontinued due to neutropenia)	None	Methotrexate (discontinued due to elevated liver enzymes)	None	None	None
**Current Immunosuppression**	Methotrexate	Methotrexate	None	Methotrexate	Azathioprine	None	Azathioprine	None
**Ocular Surgical History**	Cataract surgery OU + tube shunt OU	None	PPV, lensectomy, synechiolysis, membrane peel, retinectomy, endolaser, silicone oil OU	Cataract surgery OU + diode CPC OU	Cataract surgery OD, tube shunt OU	Cataract surgery OS + synechiolysis OD, diode CPC OD	Tube shunt OU	None

OD = right eye; OS = left eye, OU = both eyes; PPV = pars plana vitrectomy; CPC = cyclophotocoagulation.
